# Evolving therapeutic role of bisphosphonates in multiple myeloma

**DOI:** 10.1038/sj.bjc.6602694

**Published:** 2005-07-12

**Authors:** A U Ural, F Avcu

**Affiliations:** 1 Department of Hematology, Gulhane Military Medical Academy, Ankara, Turkey; 2 Department of Medical Research Center, Gulhane Military Medical Academy, Ankara, Turkey

**Sir**,

We read the interesting article by Morgan and Davies (2005) on the novel therapeutic strategies such as thalidomide and derivatives, proteasome inhibitors, and targeted therapy in the maintenance setting or in the induction phase of myeloma treatment prior to high-dose therapy (HDT). They emphasised the importance of the characterisation of the myeloma genome before selecting a treatment, the diagnosing of myeloma at an early stage to deliver the novel treatment in the natural history of their disease, and the establishment of the response rates and lengths of remission before novel combinations could be compared with HDT. However, there is no mention about the novel antitumour activity of bisphosphonates (BPs) besides its inhibiting effects on bone resorption in multiple myeloma (MM).

As is well known, BPs are used to treat osteoclast-mediated bone diseases, including osteoporosis, Paget's disease, hypercalcemia of malignancy, bone metastases, and bone disease associated with MM (Berenson *et al*, 1996; Jantunen, 2002). Current views suggest that BPs may affect differentiation and recruitment of osteoclast precursors (Hughes *et al*, 1989) or alter the capability of mature osteoclasts to resorb bone by altering the permeability of the osteoclast membranes to small ions (Sato *et al*, 1991). As a member, the more potent nitrogen-containing group of BPs, zoledronic acid, inhibits protein prenylation, thus affecting osteoclast function and survival. As protein prenylation is required by all cells, not just osteoclasts, the possibility that nitrogen-containing BPs could also affect the viability of tumour cells arises (Green, 2003). Several studies have clearly demonstrated that BPs are cytostatic to tumour cells *in vitro*, induce apoptosis, inhibit cell adhesion and interfere with the metastatic process (Aparicio *et al*, 1998). We have recently demonstrated that zoledronic acid induced antiproliferative and apoptotic effects on MM cell lines *in vitro* by activating protein kinase C and increasing extracellular calcium concentration, and these effects augmented with dexamethasone and thalidomide addition to zoledronic acid (Ural *et al*, 2003). Bisphosphonates may exert their antimyeloma effect by inhibiting release of bone marrow-derived growth factor, such as transforming growth factor *β* and insulin-like growth factor into marrow, by inducing apoptosis of MM cells, by downregulating production of interleukin 6 from bone marrow stroma, and by stimulating *γδ* T-cell-mediated antiplasma cell activity in the marrow (Mundy and Yoneda, 1998; Kunzmann *et al*, 2000). Therefore, zoledronic acid may augment *in vivo* the therapeutic action of dexamethasone and thalidomide through direct effects on myeloma cells as well as by inhibition of paracrine and autocrine signals by bone marrow stromal cells (Corral *et al*, 1996; Tassone *et al*, 2000). In this context, the results of clinical studies have suggested that BPs may reduce tumor burden and may improve survival of patients with MM (Berenson *et al*, 1998; Mundy and Yoneda, 1998). In addition, objective remission or inhibition of disease progression has been reported in patients with MM who underwent pamidronate treatment alone (Dhodapkar *et al*, 1998). In another study of ours, we demonstrated that zoledronic acid was able to increase disease-free survival in the pristane-induced plasmacytoma, a model with no direct bone involvement, in BALB/c mice model (Avcu *et al*, 2005). In this study, zoledronic acid treatment markedly impeded intraperitoneal plasmacytoma development. It also decreased tumour burden and extramedullary tumour growth in mice. Moreover, in contrast to many other animal tumours studied, which used high doses of BPs (Guenther *et al*, 2002; Croucher *et al*, 2003), the zoledronic acid dose of 20 *μ*g kg^−1^ week^−1^ s.c., which was efficacious in the murine plasmacytoma model is approximately equivalent to the approved clinical dose for the treatment of the skeletal complications of MM (4 mg every 3–4 weeks i.v.). All these results raise the possibility that nitrogen-containing BPs, such as zolederonic acid, with their direct antitumour effects, may be valuable adjuncts to the novel therapeutic strategies in the treatment of MM.
